# Tumor‐derived microparticles in tumor immunology and immunotherapy

**DOI:** 10.1002/eji.202048548

**Published:** 2020-10-28

**Authors:** Jingwei Ma, Huafeng Zhang, Ke Tang, Bo Huang

**Affiliations:** ^1^ Department of Immunology, Tongji Medical College Huazhong University of Science & Technology Wuhan P. R. China; ^2^ Department of Pathology, School of Basic Medicine, Tongji Medical College Huazhong University of Science & Technology Wuhan P. R. China; ^3^ Department of Biochemistry & Molecular Biology, Tongji Medical College Huazhong University of Science & Technology Wuhan P. R. China; ^4^ Department of Immunology & National Key Laboratory of Medical Molecular Biology, Institute of Basic Medical Sciences Chinese Academy of Medical Sciences (CAMS) & Peking Union Medical College Beijing P. R. China; ^5^ Clinical Immunology Center CAMS Beijing P. R. China

**Keywords:** DCs, tumor cell‐derived microparticle, TAMs, tumor vaccine, tumor immunotherapy

## Abstract

Microvesicles or microparticles, a type of cytoplasm membrane‐derived extracellular vesicles, can be released by cancer cells or normal cell types. Alteration of F‐actin cytoskeleton by various signals may lead to the cytoplasm membrane encapsulating cellular contents to form microparticles, which contain various messenger molecules, including enzymes, RNAs and even DNA fragments, and are released to extracellular space. The release of microparticles by tumor cells (T‐MPs) is a very common event in tumor microenvironments. As a result, T‐MPs not only influence tumor cell biology but also profoundly forge tumor immunology. Moreover, T‐MPs can act as a natural vehicle that delivers therapeutic drugs to tumor cells and immune cells, thus, remodeling tumor microenvironments and resetting antitumor immune responses, thus, conferring T‐MPs a potential role in tumor immunotherapies and tumor vaccines. In this review, we focus on the double‐edged sword role of T‐MPs in tumor immunology, specifically in TAMs and DCs, and emphasize the application of drug‐packaging T‐MPs in cancer patients. We aim to provide a new angle to understand immuno‐oncology and new strategies for cancer immunotherapy.

## Graphical abstract

Tumor cell‐derived MPs (T‐MPs) not only directly stimulate angiogenesis, invasion, and metastasis at primary tumor sites through contained molecules, but also contribute to the premetastatic niche formation by reprogramming macrophages. T‐MPs activate antitumor immunity, thus, conferring a potential role of T‐MPs in tumor immunotherapy and tumor vaccines. Furthermore, T‐MPs can act as a natural vehicle that delivers therapeutic drugs to tumor cells and immune cells, thus, remodeling tumor microenvironments and resetting antitumor immune responses.

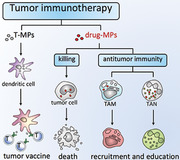

## Introduction

Cells are capable of generating several types of extracellular vehicles (EVs) including exosomes, microparticles (MPs) (or microvesicles), and apoptotic bodies [1]. These EVs are classified based on their size and the formed mechanism [2]. Exosomes are generated in multivesicular bodies with small size (30‐100 nm); they are released from the endosomes upon fusion with the plasma membrane to the extracellular space [3]. In response to various stimuli, upon the release of Ca^2+^ from the endoplasmic reticulum, cells change their cytoskeletal structure and lead to the encapsulation of cytosolic components by the plasma membrane, followed by the release of vesicles into the extracellular space. Such subcellular vesicles with a size around 100‐1000 nm are coined MPs [2, 4, 5]. In 1967, Wolf first observed MPs from platelets and described them as procoagulant “dust” [6]. In fact, MPs can be released from almost all cell types and their function depends largely on the state of their originated cells under physiological or pathological conditions [7]. MPs not only contain various molecules, including messenger molecules, enzymes, RNAs, and even DNA fragments, but also transfer these bioactive molecules from donor cells to recipient cells [5, 8]. Due to the plasma membrane derivation, several membrane‐related proteins have been proposed to be MP‐specific, such as phosphatidylserine (PS), selectins, integrins, matrix metalloproteinase (MMP), CD40, ADP‐ribosylation factor 6 (ARF6), and Rho family members [9]. The presence of MPs in wide spectrum of body fluids such as blood (plasma or serum), urine, cerebrospinal fluid (CSF), bile, ascites, and saliva of patients potentiates them as useful prognostic and predictive biomarkers, even as multiple therapeutic means [10, 11]. Described methods for MP isolation include step‐wise centrifugation which removes large cellular debris, followed by ultracentrifugation (14 000 *g*) to pellet the nano‐sized vesicles [12].

In tumor microenvironments (TME), the generation of tumor cell‐derived MPs (T‐MPs) might be a very common event, implying that T‐MPs are probably involved in tumorigenesis. Various damage‐associated molecular patterns (DAMPs), hypoxia, nutrient deficiency, chemotherapy and radiotherapy, either stimulate tumor cells or cause their death, leading to releasing T‐MPs [13, 14]. Researchers’ understanding of the role of T‐MPs in tumorigenesis is increasing vastly. T‐MPs not only directly stimulate angiogenesis, invasion, and metastasis at primary tumor sites through contained molecules [15, 16, 17], but also contribute to the premetastatic niche formation by reprogramming macrophages [18]. It has been reported that T‐MPs taken up by immune cells, such as macrophages, may lead to inhibition or alteration of antitumor immune responses [19, 20, 21]. Thus, T‐MPs are involved in tumorigenesis at multiple levels and might also be useful in disease staging. On the other hand, T‐MPs also activate the cGAS‐STING signaling, an important pathway for antitumor immunity, thus, conferring a potential role of T‐MPs in tumor immunotherapy and tumor vaccines [22]. Furthermore, considering the similarities of size, structure, and vector function between T‐MPs and artificial nanoparticles, it is reasonable to use T‐MPs as endogenous natural vehicles that deliver therapeutic drugs to target tumor cells or even immune cells such as tumor‐associated macrophages (TAMs), thus, remodeling TME and resetting the antitumor immune responses.

This review focuses on the double‐edged sword role of T‐MPs in tumor immunology and emphasizes the applications of drug‐packaging tumor MPs in the clinic. We aim to provide a new angle to understand immuno‐oncology and new strategies for cancer immunotherapy.

## Tumor‐promoting effects of T‐MPs by resetting macrophages

Macrophages display remarkable plasticity and exert different functions in different microenvironments [23]. TAMs, when expressing a M1‐like proinflammatory phenotype, play an antitumor role by producing substantial nitric oxide and other mediators [24]. In contrast to proinflammatory macrophages, M2‐like TAMs not only mediate tumor immunosuppression, angiogenesis, and metastasis but also enhance chemotherapeutic drug resistance, cancer cell survival, and cancer stem cell development [25]. A variety of factors in TME, including extracellular matrix components, IL‐10, CSF‐1, and chemokines (CCL2, CCL18, CCL17, and CXCL4), are able to educate the differentiation of macrophages toward M2‐like TAMs [26]. In addition to these factors, T‐MPs might also play a very important role in the induction of M2‐like TAMs [21]. First, in the TME exist abundant stimulating signals, such as DAMPs and apoptotic cues, such as hypoxia, which induce tumor cells to continually release MPs. Typically, tumor cells commonly express TLR4 and the activation of TLR4 signaling leads to the cytoskeletal alteration and subsequent, the generation of T‐MPs through cellular membrane shedding [13, 27]. Second, macrophages as professional phagocytes are capable of taking up T‐MPs highly efficiently [14, 21]. And third, T‐MPs can promote not only mouse macrophages but also monocytes to polarize toward anti‐inflammatory M2 phenotype [21]. Moreover, T‐MPs seem to be able to promote the macrophages to proliferate at least in vitro culture condition [21]. Although activated macrophages are considered as mature cells, they seem to still have the ability to proliferate [28, 29]. Treatment with T‐MPs leads to the increase of S/G1 cell cycle in M2‐like macrophages, which in turn induces the apoptosis of proinflammatory ones. Intriguingly, neither immune cell‐derived nor normal tissue cell‐derived MPs can induce M2‐like macrophage development [21]. Arginase 1 is a typical marker for M2‐like macrophages that could be induced by tumor lysates and lactic acid [30]. By comparison, T‐MPs have shown a stronger induction of arginase 1 than tumor lysates or lactic acid [21]. Besides arginase 1, other M2‐like markers, such as CD206, CD301, and IL‐10, are also upregulated in macrophages by T‐MPs; in contrast, the expression of proinflammatory factors, such as iNOS, TNF‐α, IL12, are found to be downregulated by T‐MPs [21]. Thus, T‐MPs may function as a common pathway to induce the development of macrophages toward a M2‐like phenotype in TME.

T‐MPs are produced by primary tumor cells and exert their effects in the tumor microenvironment. However, these T‐MPs can also function at distant organs delivered by the circulation [18]. Capillary blood vessels are physiologically permeable to nanoparticles up to 5 to 12 nm in size [31], which prevent the entry of T‐MPs (100‐1000 nm) from circulation to parenchymal tissues. However, the lungs are an exception in that the preexisting apertures in the basal lamina of alveolar capillaries and epithelium range from 0.3 to 3 μm in size [32], which allow MPs to freely cross‐membrane barriers and enter alveoli and even, enter interstitial parenchyma. Whether and how the normal lung macrophages are affected by these invading T‐MPs? In mouse models, it is demonstrated that T‐MPs can build a tumor premetastatic niche for primary tumor cell colonization and growth in the lungs [18]. This is because upon entry into the lungs, circulating T‐MPs are taken up by lung macrophages, resulting in the macrophages to release CCL2. CCL2 recruits peripheral monocytes to the lungs where the monocytes are differentiated into macrophages and produce IL‐6 [18]. On the other hand, T‐MPs induce macrophages to produce VEGF, thus, increasing endothelial permeability and leading to fibrin deposition. IL‐6 and fibrin deposition function as chemical and mechanical signals, respectively, to facilitate stem cell‐like tumor cell development in the lungs [18].

**Figure 1:**
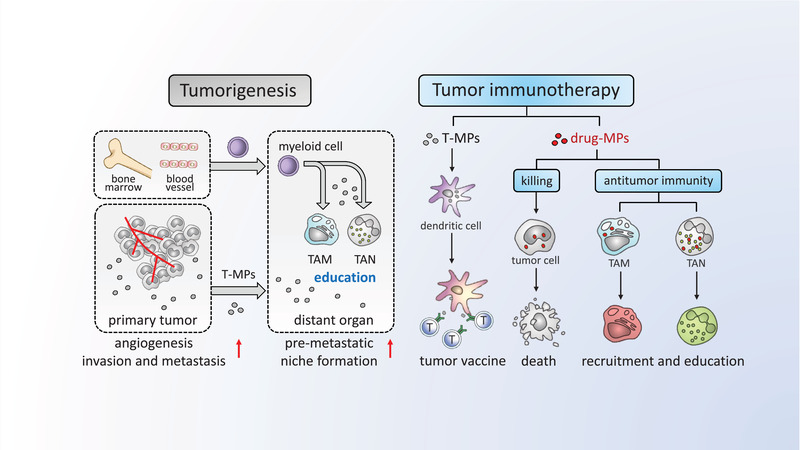
The double‐edged sword role of T‐MPs in tumor immunology and tumor immunotherapy. The release of microparticles by tumor cells is a very common event in tumor microenvironments. T‐MPs not only directly stimulate angiogenesis, invasion, and metastasis at primary tumor sites through contained molecules, but also contribute to the premetastatic niche formation by reprogramming macrophages. On the other hand, T‐MPs also activate antitumor immunity, thus, conferring a potential role of T‐MPs in tumor immunotherapy and tumor vaccines. Furthermore, T‐MPs can act as a natural vehicle that delivers therapeutic drugs to tumor cells and immune cells, thus, remodeling tumor microenvironments and resetting antitumor immune responses. T‐MPs, tumor cell‐derived MPs; drug‐MPs, drug‐packaging T‐MPs; T, T cell; TAM, tumor associated macrophage; TAN, tumor associated neutrophil.

M1‐like macrophages are generally linked to inflammation and M2‐lke macrophages are considered to play an anti‐inflammatory effect [33]. It has been reported that T‐MPs carry hyaluronan and the latter is able to induce IL‐10 production in human macrophages via the PI3K/AKT/mTOR signaling pathway [34]. In line with the M2‐like phenotype, T‐MP‐educated macrophages display an immunosuppressive function [21]. Surprisingly, mouse and human macrophages can also be induced by T‐MPs to release proinflammatory cytokine IL‐1β, thus, remodeling tumor inflammation and immunosuppression and promoting cancer development [14]. T‐MPs contain varieties of noncoding RNAs. These RNAs trigger the activation of TLR3, leading to the upregulation of IL‐1β precursor. On the other hand, T‐MPs activate lysosomal Ca^2+^ channels of macrophages, leading to Ca^2+^ release to the cytosol [14]. In turn, this released Ca^2+^ triggers mitochondrial reactive oxygen species (ROS) production to activate the NLRP3 inflammasome, leading to the cleavage of IL‐1β [14]. Taken together, tumor cell‐derived MPs have versatile abilities to reset macrophages as tumor culprits (Fig. 1).

## DCs are activated by T‐MPs

Like macrophages, DCs are also capable of taking up T‐MPs [22, 35]. In TME, DCs capture tumor antigens for presentation at draining lymph nodes (DLN). However, tumor‐originated factors impede this process by suppressing DC activation. As a result, DCs cannot upregulate chemokine receptor CCR7 for migration to DLNs by CCL19 and CCL21 [36]. Or even DCs arrive at DLN, where they induce T‐cell anergy due to insufficient costimulatory signal [37]. Tumor‐associated factors, such as VEGF, TGF‐β, IL‐10, PGE2, and IDO, can profoundly affect the nature of DCs by inhibition of their activation [38, 39]. Such tumor‐educated DCs either enter paralysis or become a tumor accomplice by inducing regulatory T‐cells or releasing angiogenic factors in TME [40]. Considering the effect on macrophages, it is reasonable to speculate that DCs are similarly educated into tumor‐promoting ones by T‐MPs, but the answer is no. T‐MPs do not suppress mouse DCs; in contrast, they activate DCs for antigen presentation [22, 35]. Following uptake of T‐MPs, cGAS‐STING, a key innate signaling pathway, is triggered in mouse DCs. This is ascribable to DNA fragments within T‐MPs. Apoptotic tumor cells might release DNA fragments to the cytoplasm, leading to the entry of DNA fragments into T‐MPs during their formation. Both mitochondrial and genomic DNA fragments are found in the T‐MPs. Considering the bacterial origin, it might be mitochondrial DNA that play the major role in the activation of the cGAS‐STING pathway. This cGAS‐STING signaling leads to IRF3 and IRF7 activation via TBK1 and subsequent production of type I IFN [22]. In line with this result, DCs upregulate the expression of CD80, CD86, MHC class II, IL‐12, and even IFN‐γ, suggesting that DCs are indeed activated by T‐MPs [22] (Fig. 1).

## Different consequence of T‐MPs on macrophages and DCs is explained by lysosomes

Next, why do the same T‐MPs result in a different consequence between macrophages and DCs? The answer might lie in lysosomes. Lysosomes are the cellular compartment for the degradation of biological macromolecules [41]. Beyond the canonical role in cellular waste disposal, lysosomes also play important roles in nutrient sensing, metabolism, membrane repair, and immune cell signaling [42]. A unique feature that distinguishes the lysosome from other organelles is the acidic lysosomal pH, which renders lysosomal hydrolases stable and active, regulates the dynamics of lysosomal membrane proteins, and facilitates vesicular trafficking [43]. In addition to H^+^, luminal lysosomal Ca^2+^ is also indispensable for lysosome function exertion [44]. Both mouse macrophages and DCs take up T‐MPs through phagocytosis, which delivers T‐MPs to endosomes [14, 35]. The latter then fuses with lysosomes, resulting in T‐MP degradation. However, the process might be not that simple. The influence of T‐MPs on lysosomes is totally different between macrophages and DCs. A typical feature for lysosomes is the pH value. Lysosomes physiologically have a low pH value around 4.5‐5.0 [45]. T‐MPs can increase the lysosomal pH of DCs [35]; in contrast, they decrease the lysosomal pH of macrophages [14]. This inconsistence is intriguing and probably reflects the different roles even though macrophages and DCs both belong to phagocytes. Upon taking up exogenous materials, DCs are professional APCs, which increase lysosomal pH for antigen cross‐presentation [46, 47]; however, macrophages are professional phagocytes, which decrease lysosomal pH to better degrade wastes [48]. Enzymes in lysosomes continually degrade materials at the expense of consuming protons; on the other hand, vacuolar‐type H^+^‐ATPase (V‐ATPase) on lysosomal membranes continually pumps protons from the cytosol into the lysosomal lumen, thus, maintaining the low lysosomal pH value [49]. Lysosomal V‐ATPase consists of V0 and V1 domains, each with multiple subunits [50]. The uptake of T‐MPs does leads to the upregulation of subunits V0a2 and V0a3 on lysosomal membrane of macrophages, thus, increasing the pumping of protons and subsequently decreasing lysosomal pH value [14]. However, the uptake of T‐MPs does not alter the expression of V‐ATPase subunits in DCs. Intriguingly, enzyme NADPH oxidase 2 (NOX2, previously known as gp91phox) is activated by T‐MPs, and catalyzes the generation of superoxide anion that is quickly converted into hydrogen peroxide by consuming protons, leading to increasing pH value in DCs, consistent with a previous report [51]. Importantly, T‐MPs do not affect the NOX2 system in macrophages [35]. How does this lysosomal pH alteration influence the phenotype and function of macrophages and DCs, respectively? To date, the answer for this question remains unclear. Intriguingly, in DCs, the increased pH value by T‐MPs is triggered by increased ROS, which in turn activates one lysosomal Ca^2+^ channel, leading to the release of lysosomal Ca^2+^. The released Ca^2+^ then activates transcription factor EB (TFEB), a lysosomal master regulator and an important transcription factor, which directly binds to the promoters of CD80 and CD86 genes, promoting gene expression [35]. Lysosomes are a critical organelle of a cell in that they are the key sensor of energy and nutrient metabolism for a cell [45]. Lysosomes regulate mTOR activation through recruiting GTP‐binding Rheb to lysosomal membrane. With environmental nutrient input, lysosomal membrane Rag‐Ragulator‐V‐ATPase complex is activated, which promotes the formation of mTORC1 [52]. Intriguingly, lysosomes also regulate nutrient insufficiency. The unavailability of glucose leads to the uncouple of FBP with aldolase and allows aldolase to bind Axin‐LKB1 complex and further bind to the V‐ATPase‐Ragulator. Subsequently, LKB1 phosphorylates and activates AMPK [53]. Thus, lysosomes are the key site where cellular metabolism is regulated [53]. In this regard, how T‐MPs regulate the metabolism of macrophages and DCs are worthy of investigation.

## T‐MPs act as potential cancer vaccines

Success of a tumor vaccine relies on the ability to provide antigen‐presenting cells with both tumor antigens and immunostimulatory signals, leading to efficacious tumor‐specific T‐cell immune responses [22]. Given the high availability of tumor antigens, the great challenge of tumor vaccines is how to form a suitable Th1‐like microenvironment for T‐cell activation and differentiation. For example, whole tumor cells contain both mutated neoantigens and nonmutated tumor‐associated antigens, which is likely to overcome the potential immune escape and resistance. However, tumor cell‐derived cytokines (e.g., VEGF, IL10, and TGF‐β) and inhibitory factors (e.g., galectin‐1, indoleamine 2,3‐dioxygenase, and lipid mediators) are ready to suppress DC maturation and T‐cell activation [54, 55]. Currently, synthetic biomaterials are explored to deliver tumor antigens to DCs with innate stimulating signals [56]. Compared to synthetic biomaterials, T‐MPs as natural counterparts have versatile merits for tumor vaccine development. T‐MPs are an ideal carrier to deliver antigens. For example, MPs released from *Listeria monocytogenes*‐infected macrophages effectively transfer *L. monocytogenes* antigens to DCs [57]. T‐MPs also carry tumor antigen repertoires and present them to DCs [35]. Furthermore, T‐MPs generated from UV‐irradiated tumor cells may contain stimulatory molecules, such as DNA fragments, thus, stimulating the production of type I IFNs, IL‐12, and IFN‐γ by DCs [35]. It has been reported that type I interferons play a critical role in CD8^+^ T‐cell priming, and both IL‐12 and IFN‐γ facilitate antitumor T‐cell activation [58]. Thus, although tumor cells contain various immunosuppressive factors, TMPs seem to contain exceeding immunostimulating factors, resulting in generation of innate immune signals in DCs.

Success of tumor vaccines rely on the cross‐presentation of DCs. Cross‐presentation refers to the presentation of peptides derived from extracellular proteins including those from internalized proteins, microvesicles, or dying cells [59]. Upon endocytosis/phagocytosis, extracellular proteins are delivered into endosomes/phagosomes, where exogenous antigens are degraded into antigenic peptides to bind MHC class I molecule for the cross‐presentation to the plasma membrane [59]. Alternatively, exogenous proteins can be translocated from endosomes/phagosomes to the cytoplasm, where they undergo degradation by the proteasome. This altered cross‐presentation is called the cytosolic pathway [59]. DCs use phagocytosis to take up T‐MPs [35]. Within phagosomes, T‐MP‐contained tumor antigens are degraded to tumor antigenic peptides. This process is regulated by T‐MP‐caused pH increase through NOX2‐mediated ROS production and proton consumption [35]. A transient alkalization is necessary to avoid overdegradation of tumor antigens into shorter peptides or even single amino acids [35, 60]. Specialized DCs like CD103^+^DCs, BATF3^+^ DCs, or Langerhans cells are considered to biasedly conduct cross‐presentation [61, 62]. This might be due to the existence of a suitable machinery to process class I antigen in these DCs; however, T‐MPs enhance the effectiveness of this machinery through regulating lysosomal NOX2‐ROS in DCs [35]. In addition, DCs could use the endogenous MHC class I antigen presentation pathway for cross‐presentation [63]. Earlier studies have shown that the cross‐presentation of exogenous antigens appears in an early endosomal compartment through a TLR4‐MYD88‐ dependent recruitment of TAP1/TAP2 to the early endosome [64]. Whether and how DCs deal with T‐MPs in the early endosomal compartment for antigen presentation is worthy of investigation. Currently, the exploration of T‐MPs as tumor vaccine is further extended by nanotechnology. Nano‐Fe_3_O_4_ is encapsulated in T‐MPs to prepare Fe_3_O_4_/T‐MPs, which are tethered with CpG‐loaded liposomes on the surface to generate a vaccine of Fe_3_O_4_/T‐MPs‐CpG/Lipo. This nano‐modified T‐MP vaccine elicits strong antitumor T‐cell immune response by triggering DC antigen cross‐presentation [65]. In addition, studies from the Mooney's group have explored two mechanical approaches (extrusion and sonication, respectively) to produce subcellular vesicles, and they demonstrated that adjuvant (CpG)‐loaded such microvesicles can present tumor antigens to DCs and induce antitumor T‐cell response [66]. However, such subcellular vesicles without CpG loading do not generate innate signal(s) to stimulate DCs, which is different from T‐MPs that can activate cGAS/STING. Another distinctive feature of T‐MP vaccine is its oral vaccination. Despite the subcutaneous or intravenous injection as the common means for vaccination, recent advances in mucosal immunity provide new opportunities to explore the oral route for prophylactic and therapeutic vaccination. We have demonstrated that orally administered mouse tumor cell‐derived MPs can interact with ileac intestinal epithelial cells and activate the NOD2 signaling for CCL2 production and the recruitment of CD103^+^ DCs, leading to mucosal and systemic antitumor T‐ cell immunity in mice [67]. Parenky’ group further studied the antitumor effect of oral MP vaccine in combination with two clinical drugs (cyclophosphamide and GM‐CSF) in a murine prostate cancer model [68]. They demonstrated a fivefold reduction in tumor volume and a significant reduction in regulatory T‐cells compared to nonvaccinated mice. These findings provide evidence that T‐MPs might be used as an oral vaccine against cancers. Despite these advantages of T‐MPs as a new tumor vaccine platform, it is unclear to what extent natural T‐MPs contain tumor antigen materials. Although MHC class I molecule might be downregulated in tumor cells, T‐MPs have been found to express decent MHC class I, hinting the binding of tumor antigen peptide [69]. Certainly, T‐MPs may also contain tumor antigens with undegraded polypeptide chain. In addition, tumor antigen‐encoding mRNAs can be included by T‐MPs and translated in DCs [22, 66]. Thus, T‐MP‐contained tumor antigen materials may be presented in a different manner. In addition, comparing with apoptotic tumor cells or the lysates, tumor materials are much easier to be taken up by DCs [22, 66], hinting an effective transfer of tumor antigens from T‐MPs to DCs. Considering the potential application of T‐MPs for cancer vaccines, the extent of T‐MP‐contained tumor antigen materials needs to be elucidated.

## Drug‐packaging T‐MPs remodel TME

TME profoundly influence immunotherapy and clinical outcomes [70, 71]. Remodeling the tumor microenvironment is an emerging strategy for improved immunotherapy [72]. Modulation of stromal cells is a common strategy in ameliorating TME; however, targeting tumor cells especially for tumor‐repopulating cells (TRC) is an alternative strategy for cancer immunotherapy. T‐MPs, due to their capacity to package large amount of drug molecules, lipid raft‐like membrane structure and safety (autologous components), are an ideal carrier to deliver chemotherapeutic drugs to tumor cells, leading to effective killing of tumor cells [73, 74]. An important issue is how the drug‐packaging T‐MPs (drug MPs) enter tumor sites. Malignant pleural effusion (MPE) or malignant ascites are common metastatic tumors, which are administrated with drainage tube for palliative treatment. Thus, drug‐MPs can be delivered along the tube into malignant fluids where tumor cells live. The second means to deliver drug‐MPs is the direct injection into superficial solid tumors such as melanoma. However, for most solid tumors, drug‐MPs can be delivered via intravenous injection. Physiological capillary gaps are around 5‐8 nm [75], however, tumor capillary permeability can be around 780 nm [76, 77], thus, preventing T‐MPs (100‐1000 nm in size) from reaching normal tissues but allowing them into tumor parenchyma. Such delivery efficiency has been improved recently by enhancing the physical softness of T‐MPs [76, 77]. Intriguingly, the drug‐packaging human tumor cell‐derived MPs prefer to target TRCs, which are highly tumorigenic and can grow colonies in 90 Pa soft 3D fibrin gels [69, 79]. This is ascribe to the mechanical softness of TRCs, leading to easy deformation and uptake of drug MPs [69]. Following the uptake, drug MPs enter lysosomes whereby they recruit motor protein dynein through activating Rab7, thus, pushing lysosome migration along microtubule to the nearby of the nucleus where the lysosomes download the drug molecules and allow them enter the nucleus through nuclear pores [69, 80]. Such killing of TRCs and differentiated tumor cells by drug MPs undoubtedly ameliorates the immunosuppressive microenvironment of tumor.

In addition to targeting tumor cells, drug‐MPs also target TAMs, a key player that contributes tumor immunosuppression, cancer stemness, and metastasis [81]. As discussed above, T‐MPs promote M2‐like TAM development [21]. Drug‐packaging T‐MPs, however, reset TAMs to develop toward the M1‐like phenotype. Such difference is due to a very low amount of chemotherapeutic drug in the T‐MPs [81]. A low drug content may not result in a direct cytotoxicity to the cells but acts as a lysosomal regulator upon T‐MPs entering lysosomes, thus, reprogramming macrophages’ phenotype. Currently, this reprogramming mechanism is underlying investigation. Such drug MPs combining low‐dose irradiation can effectively curtail TRCs and relieve TRC‐mediated suppressive configuration of TAMs. The resultant, M1‐like macrophages remodel tumor microenvironment by decreasing immunosuppressive cells and increasing T‐cell infiltration, leading to effective antitumor T‐ cell immunity [81]. Thus, drug MPs provide a novel strategy to reprogram polarized macrophages from tumor promoting to tumor inhibiting, with potential clinical applications.

Neutrophils are the most abundant innate immune cells, representing 50‐70% of all leukocytes and more than 10^11^ neutrophils can be produced daily in our body [82], implying that the same number of neutrophils undergo death per day. Although neutrophils can be elicited to function as an antitumor weapon in certain cancer patients, there is strong evidence that tumor‐associated neutrophils (TANs) may be refined to become tumor accomplices in TME. Many tumor‐associated factors, such as inhibitory cytokines, hypoxia, low pH, potassium overabundance, and tumor‐associated stromal cells, can redirect neutrophils to become tumor‐promoting ones [83, 84]. Our recent studies, however, found that drug MPs highly efficiently mobilize endogenous neutrophils and arouse the intrinsic antitumor activities. The attracted neutrophils display a CD11b^+^CD15b^+^ a more mature phenotype and exert a tumor cell‐killing effect by releasing ROS and NO in TME [85].

Tumor‐infiltrating immune cells mostly are myeloid cells [86]. Such myeloid cells are usually conferred by tumor cells with an immunosuppressive phenotype. However, it might be possible for this suppressive phenotype to be reversed by drug MPs. Most myeloid cells have the phagocytotic ability to take up drug MPs and upon uptake, it can profoundly influence the biology of lysosomes, through which the phenotype and function of tumor‐infiltrating myeloid cells are altered, thus, remodeling TME MHC class II, IL‐12, and even IFN‐γ, suggesting that DCs are indeed activated by T‐MPs [22] (Fig. 1).

### Clinical application of drug‐packaging T‐MPs

Drug‐packaging human tumor cell‐derived MPs seem to have multiple virtues which potentiate the application in the clinic. First, T‐MPs are generated from cellular membranes, making them very safer and self‐friendlier; second, the preparation of drug‐MPs is simple and easy to manipulate; third, packaging MPs with drugs is a simple way and not limited by physicochemical properties of drugs [69]; and fourth, drug‐MPs target many phagocytes and can ameliorate immunosuppressive TME.

### The efficacy of drug MPs against patients’ MPE

Drug MPs can be administrated to patients through different ways. Intravenous injection may result in the delivery of drug MPs into solid tumors via the increased permeability of tumor papillary vessels. It might also be suitable to directly inject drug MPs to superficial tumors or to inject it into cavities (pleural or peritoneal cavity) where MPE or ascites exists [69, 85, 87]. Both MPE and malignant ascites are very common in advanced malignancies [88, 89]. Despite the progress in cancer treatment, the current management of malignant fluids remains palliative. For instance, the management of MPE includes pleurodesis and indwelling pleural catheters, both of which are suboptimal in terms of their efficacy and safety, along with a median survival rate ranging from 3 to 12 months [90]. Considering pleural cavity as a closed cavity and the safety of drug MPs inside, in a clinical study, six end‐stage lung cancer patients with MPE were recruited to evaluate its efficiency. Three patients were treated with intrathoracic injection of cisplatin‐packaging MPs, and the other three patients were treated with cisplatin. After a 7‐day drug‐MP treatment, more than 95% tumor cells in the malignant fluids disappeared, which could be explained by the cytotoxicity of drug MPs to tumor cells. Intriguingly, the fluid in the pleural cavity was reduced quickly [69]. An unappealing cue from the treatment is that the bloody color of malignant fluids becomes yellow overnight in most patients, hinting that drug‐MP treatment can effectively seal off the gap of the damaged endothelium [85, 87]. If it is true, how does this take place?

In H22 hepatocellular carcinoma‐induced ascites mouse model, peritoneal injection of drug‐packaging mouse tumor cell‐derived MPs result in attraction of abundant neutrophils into ascites [85]. Consistently, a large number of neutrophils in patients’ MPE have been observed following the treatment [85]. The recruited neutrophils display a mature phenotype with the expression of CD11b and CD15 and an active state with the release of ROS. By ex vivo incubation, tumor cells can be attacked by the released ROS, suggesting that in addition to direct killing, drug‐MPs may use neutrophils as an indirect way to kill tumor cells [85]. Notably, the resultant ROS can trigger neutrophil extracellular trap (NET) formation [91]. NETs are a web of fibers by chromatin and serine proteases, which trap and kill extracellular microbes and also can act as a physical barrier, preventing further spread of pathogens [92, 93]. However, NETs may also have a pathological side by exacerbating organ injury and promoting metastasis [94, 95]. Very interestingly, drug‐MP‐triggered NETs are involved in the fluid reduction. This is because the released NETs use their viscosity to attach and seal off the damaged endothelium, thus, inhibiting vessel leakage [85]. To date, two registered randomized controlled clinical trials (ChiCTR‐TRC‐14004820 and ChiCTR‐ICR‐15006304) are involved in the treatment of MPE by drug MPs. All in all, drug MPs, by virtue of their ability to recruit neutrophils, result in a robust neutrophil immune response against tumor, thus, providing insights into the versatile role of neutrophils in tumor treatments, especially in the treatment of malignant fluids [85].

### The efficiency of drug MPs in obstructive cholangiocarcinoma

Cholangiocarcinoma (CCA) is a lethal adenocarcinoma that arises from the epithelial lining of the biliary tree [96, 97]. More than 90% of CCAs emerge from the extrahepatic bile ducts, which are liable to form malignant biliary obstructions [98, 99]. No efficacious treatment option is available for obstructive CCA. Given the merits of drug‐packaging human tumor cell‐derived MPs, their use to target CCA tumor cells might be a mean to kill tumor cells and relieve the obstruction. In a recent clinical trial, injection of methotrexate (MTX)‐MPs via percutaneous transhepatic biliary drainage (PTBD) to the bile duct lumen for 7‐day treatment with patients with CCA and it could be observed a change in color of the feces to yellow [100]. Despite the strong drug resistance, CCA tumor cells are consistently very sensitive to drug MPs [100]. However, the problem is that CCA tumor cells are protected by dense extracellular matrix or even basement membrane, which prevents drug MPs from gaining access to tumor cells. Immune cells, especially neutrophils, can release enzymes, such as MMPs and neutrophil elastase (NE) to degrade stromal components, thus, loosening the stromal structure of tissues [101]. Analysis by flow cytometry of the bile drainage revealed that a large number of neutrophils are attracted to the bile following drug‐MP treatment [100]. Elastase and MMP 8 were found to be released from these neutrophils, which degrade the matrices, thus, abrogating the stromal barriers and allowing MTX‐MPs to gain access to CCA cells [100]. However, the question is how neutrophils are recruited to the bile by MTX‐MPs perfusion. Two molecules UDPG and C5, which are able to attract neutrophils [102, 103], are found in MTX‐MPs, thus, possibly recruiting neutrophils to the bile [100]. Besides, MTX‐MPs can also induce CCA cells to undergo pyroptosis through a gasdermin E (GSDME)‐dependent pathway. Nevertheless, such pyroptosis may also attract neutrophils, and the pyroptosis‐stimulated macrophages can also release factors to attract neutrophils. In addition, MTX‐MPs can stimulate macrophages to release neutrophil‐attracting factors such as CXCL1 and CXCL2 [100]. Thus, upon a simple MTX‐MP treatment, multiple neutrophil‐attracting pathways are triggered. The common consequence is that the recruited neutrophils display an antitumor trait [100].

## Conclusions and perspectives

The unprecedented success of the clinical application of programmed cell death receptor 1 (PD‐1) blockade antibodies and CAR‐T therapy marks immunotherapy of tumors comes age. However, developing a more efficient and safer immunotherapeutic is still a challenge. It is thought that a single immunotherapy is not enough to control a tumor by immune cells and that the combined strategy represents the next generation of immunotherapy of tumors. On the other hand, biomaterials, by virtues of their versatility and complex nature, present a promising approach to solve some key issues faced by current immunotherapies. Tumor cell‐derived MPs are a complex natural biomaterial with multiple immune modulatory properties. Unlike synthetic nanoparticles, T‐MPs automatically carry both DNA fragments for cGAS‐STING activation and antigens for DC presentation. Using genetic or physiochemical methods to modify parent cells, distinctive innate signals and antigens can be further selectively combined and integrated into T‐MPs, making T‐MPs more versatile and flexible. Moreover, the automatic encapsulation of chemotherapeutic drugs adds a new dimension to the application of T‐MPs, thus, intertwining nanotherapy, chemotherapy, and immunotherapy tightly. The initial success of drug‐MPs in the treatment of patients’ MPE and obstructive CCA sheds light on a wide application of T‐MPs in the clinic. Potential side effects of using drug MPs as a therapy have not been observed in patients so far. More extensive and in‐depth studies are needed to unveil the biology of T‐MPs and dissect their immunoregulation. Finally, the bold clinical studies should be designed to potentiate MP‐based immunotherapies MHC class II, IL‐12, and even IFN‐γ, suggesting that DCs are indeed activated by T‐MPs [22] (Fig. 1).

## Conflict of interest

The authors declare no commercial or financial conflict of interest.

**Abbreviations**
ARF6: ADP‐ribosylation factor 6
CCA: cholangiocarcinoma
CSF: cerebrospinal fluid
DAMPs: damage‐associated molecular patterns
DLN: draining lymph nodes
EVs: extracellular vehicles
MMP: matrix metalloproteinase
MPs: microparticles
MPE: malignant pleural effusion
NE: neutrophil elastase
NET: neutrophil extracellular trap
PS: phosphatidylserine
TAMs: tumor‐associated macrophages
TANs: tumor‐associated neutrophils
TFEB: transcription factor EB
TME: tumor microenvironments
T‐MPs: tumor cell‐derived MPs
TRC: tumor‐repopulating cells
V‐ATPase: vacuolar‐type H^+^‐ATPase
